# Peer Review of “Eye Care Service Use and Associated Health-Seeking Behaviors Among Malawian Adults: Secondary Analysis of the Malawi Fifth Integrated Household Survey 2019-2020”

**DOI:** 10.2196/57935

**Published:** 2024-04-09

**Authors:** Anonymous

**Keywords:** access to health, health service utilization, eye care use, health-seeking behavior, sociodemographic determinant, visual impairment, social support, women empowerment, education, eye care, pediatric, eye, ophthalmology, visual, ECU, eye service, utilization, Malawi, empowerment, health service use, use

*This is the peer-review report for “Eye Care Service Use and Associated Health-Seeking Behaviors Among Malawian Adults: Secondary Analysis of the Malawi Fifth Integrated Household Survey 2019-2020.”*

## Round 1 Review

### General Comments

This paper [1] is a secondary analysis of a Malawian household survey exploring associations of patients who self-reported as having used formal eye care services. It is a useful idea to use this survey data for this purpose, but the author needs to check that they are using the correct source numbers for their statistical analysis and only report the numbers actually surveyed—not the national estimated numbers derived from these.

### Specific Comments

#### Major Comments

1. “In Malawi, 3.3% of the population is blind compared to 1.01% in America [2,3].” There is no way 3% of Malawi is blind. (Half of Malawi’s population are children, so if 3% of Malawi was blind, that would be about 1 in 20 adults—not possible.) Check your references.

2. The abstract needs improving to give the definition of eye care use (ECU). In the results, it says “The prevalence of ECU was 60.6%,” which is not really a prevalence unless you give a clearer definition, for example “of those with eye symptoms, what proportion have access formal eye care services in the two weeks prior to the survey date.”

3. The sample was 28,388 adults? You cannot, then, in the results’ “Characteristics of study participants” section say there were 6 million young adults involved or that 5,660,836 (56%) of the adults were married. You also can’t say that “27,336 (0.3% of 2,734,768) complained of ocular symptoms.” This is the main problem with the report—you need to give the actual numbers of people surveyed who reported ocular symptoms—presumably 0.3% of 28,388—which is only 85 people. Thus your CIs/other statistical analyses around estimates with a sample of 85 people reporting eye symptoms will be quite different than if you extrapolate to the whole population of Malawi.

#### Minor Comments

4. “We entered the variables...”: Who is “we”? I only see one author

5. “Sort care” should be “sought care”: This is used 5 times in the paper so should be changed at all uses

6. There are some random capital letters in various places: “that In Malawi”—why has “In” got a capital?

## Abbreviations

ECU: eye care use

## References

[1] Mzumara T, Kantaris M, Afonne J (2024). Eye care service use and associated health-seeking behaviors among Malawian adults: secondary analysis of the Malawi Fifth Integrated Household Survey 2019-2020. JMIRx Med.

[2] Kalua K, Lindfield R, Mtupanyama M, Mtumodzi D, Msiska V (2011). Findings from a rapid assessment of avoidable blindness (RAAB) in Southern Malawi. PLoS One.

[3] Flaxman AD, Wittenborn JS, Robalik T (2021). Prevalence of visual acuity loss or blindness in the US: a Bayesian meta-analysis. JAMA Ophthalmol.

